# Design and Implementation of an On-Chip Low-Power and High-Flexibility System for Data Acquisition and Processing of an Inertial Measurement Unit

**DOI:** 10.3390/s20020462

**Published:** 2020-01-14

**Authors:** Zhenyi Gao, Bin Zhou, Yang Li, Lei Yang, Xiang Li, Qi Wei, Hongyang Chu, Rong Zhang

**Affiliations:** 1 Engineering Research Center for Navigation Technology, Department of Precision Instrument, Tsinghua University, Beijing 100084, China; gaozy17@mails.tsinghua.edu.cn (Z.G.); yang-li16@mails.tsinghua.edu.cn (Y.L.); li-x07@mails.tsinghua.edu.cn (X.L.); chuhy19@mails.tsinghua.edu.cn (H.C.); 2 College of Information Science and Engineering, Shandong Agricultural University, Tai’an 271018, China; yanglei@sdau.edu.cn

**Keywords:** IMU, signal processing, SoC, low power, miniaturization

## Abstract

For signal processing of a Micro-Electro-Mechanical System (MEMS) Inertial Measurement Unit (IMU), a digital-analog hybrid system-on-chip (SoC) with small area and low power consumption was designed and implemented in this paper. To increase the flexibility of the processing circuit, the designed SoC integrates a low-power processor and supports three startup or debugging modes for different application scenarios. An application-specific computing module and communication interface are designed in the circuit to meet the requirements of IMU signal processing. The configurable clock allows users to dynamically balance computing speed and power consumption in their applications. The chip was taped out under SMIC 180 nm CMOS technology and tested for performance. The results show that the chip’s maximum running frequency is 105 MHz. The total area is 33.94 ${\mathrm{mm}}^{2}$. The dynamic and static power consumption are 0.65 mW/MHz and 0.30 mW/MHz, respectively. When the system clock is 25 MHz, the dynamic and static power consumption of the chip is 76 mW and 66 mW, and the dynamic and static power consumption of the FPGA level are 634 mW and 520 mW. The results verify the superiority of the application specific integrated circuit (ASIC) solution in terms of integration and low power consumption.

## 1. Introduction

The Inertial Measurement Unit (IMU) is a group of sensors for measuring inertial data and typically contains three gyroscopes and three accelerometers [1,2]. Gyroscopes can be used to measure three-axis rotation angle or angular velocities, and accelerometers are used to measure three-axis acceleration. The inertial data output by IMU can be used for navigation, attitude calculation, etc., and thus has been widely used in aerospace, military, vehicle and other fields [3,4,5]. In practical applications, the IMU is always implemented under the MEMS technology, which makes the IMU have the characteristics of small size, low weight, low cost, and low power consumption. However, the accuracy of the MEMS IMU is relatively low compared with mechanical and optical gyroscopes and accelerometers, and the output signals are always disturbed by null drift and temperature change [6,7].

For error compensation and further processing of the data, it is necessary to perform the corresponding tasks with additional hardware. The tightly coupled navigation system mentioned in [8] is consist of a Field Programmable Gate Array (FPGA) and a Digital Signal Processor (DPS), in which the FPGA is used to conduct data acquisition (communicate with sensors, send control signals and read data) and transmission (data processing and communication with other equipment), and the DSP is used to execute the navigation algorithm. Another navigation system based on an IMU mentioned in [9] has a Micro Control Unit (MCU) to implement sensor configuration and data acquisition, and a DSP to run a signal processing algorithm. For more general IMU data acquisition and processing systems, there is no need to provide a definitive execution algorithm but rather an interface or other means to support other data processing algorithms. The system in [10] is an example. It solved the synchronization problems when data acquiring and accomplished redundant design for the system. The above-mentioned systems are capable of data collection and processing and can execute complex algorithms, but the performance of discrete devices in terms of miniaturization and low power consumption is weaker than integrated solutions.

For engineering applications based on an IMU, data acquisition and processing algorithm can be integrated on one device to further improve system functionality. The mobile motion capturing device based on an IMU designed in [11] is a typical example of data acquisition and algorithm processing implemented in an FPGA. The proposed design is a SoC and is consist of data transfer interfaces for hardware accelerate and a common processor in the FPGA for executing data processing algorithm. A SoC contains both dedicated hardware modules to efficiently perform tasks that are always required, as well as a processor that flexibly implements circuit parameter configuration and processing algorithms. Thermal calibration is another application for processing the data from an IMU with a SoC. The prototype in [12] is based on an FPGA with a dual-core processor to satisfy the demands of calibration algorithm and navigation algorithm. For general purpose processing platforms, dedicated modules and interface circuits can be designed and configured to increase the flexibility of the overall system [13,14]. The purpose in [13] is to integrate the inertial navigation system based on a SoC platform, in which data acquisition and processing modules were designed and implemented on an FPGA. The design in [14] is also general platform for data acquisition and processing, and the aim of this work is to develop a low-cost system with components easy to find in actual market. An FPGA is an integrated platform, but due to the pre-wiring implementation solution, an FPGA cannot reach the extreme performance in terms of integration and low power consumption.

FPGA-level prototyping by other researchers confirms the possibility of implementing IMU data acquisition and processing in a SoC. At the chip level, this article discusses the design principle and implementation results of a digital-analog hybrid SoC with small area and low power consumption for signal processing of a MEMS IMU. The implementation method using integrated circuits can minimize the redundancy in the system implementation process, further improve the system integration, and reduce power consumption. The designed chip was taped out under SMIC 180 nm CMOS technology and its performance was tested. Briefly speaking, the entire system consists of a low-power consumption processor, some dedicated computing modules, and interface circuits. The processor is used for parameter configuration and algorithm execution, which improve the system flexibility. The dedicated computing module is used for hardware acceleration, and the interface circuit is used for sensor data transmission or control. The entire system supports three debugging and startup modes to meet different application scenarios. The configuration of the clock frequency is supported to achieve low-power applications that meet computing requirements. In addition, temperature compensation experiments were performed based on an IMU and the designed chip, which verified that the chip works in practical applications.

This article is organized as follows. Section 2 discusses the architecture and function of the proposed SoC. Section 3 provides implementation details of the SoC. Section 4 presents the implementation results and application experiments. Finally, Section 5 provides conclusions and future research plans.

## 2. Description for the Architecture and Function of the Proposed SoC

### 2.1. Architecture Description of the Proposed SoC

**Figure 1:**
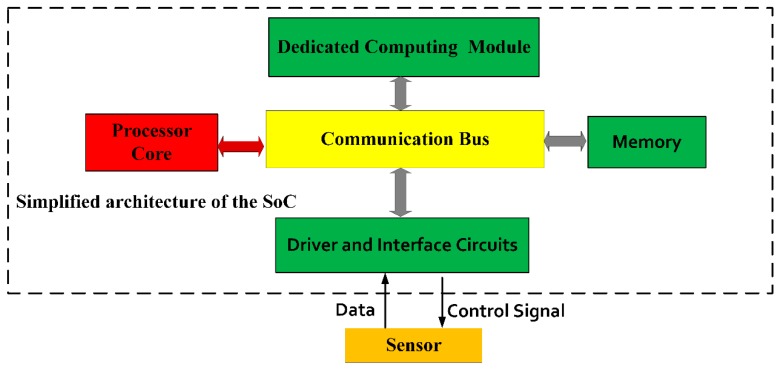
Simplified architecture of the proposed system-on-chip (SoC) for data acquisition and processing of an inertial measurement unit (IMU).

Figure 1 shows the simplified architecture of the proposed SoC. The SoC consists of a processor core, dedicated computing modules, driver and interface circuits for the sensors and memory. The processor core is Cortex-M3 [15], which is an open source Intellectual Property (IP), provided by Advanced RISC Machines Ltd (ARM Ltd, Cambridge, UK). As a low-power 32-bit processor for embedded field, it is adopted in the designed solution to improve the system flexibility and low power performance. The dynamic power consumption reported in [15] is 0.141 mW/MHz at 1.8 V operating voltage. The dedicated computing module is a high speed and high precision signal generator, which receives angular signals and outputs sine signals and cosine signals. The calculation of the sine and cosine function is commonly found in some navigation algorithms [3,4,13,16], therefore implementing it in hardware and turning the corresponding software calculation into hardware calculation can improve the execution speed of the algorithm. This helps to reduce the algorithm’s requirements on the system frequency, thereby achieving lower power consumption. The memory contains on-chip Random Access Memory (RAM), Read Only Memory (ROM), and One Time Programmable (OTP) device for data and program storage, and its different functions will be described later. Drive and interface circuits are used to handle signal transmission from sensors, external circuits, and the SoC. Control signals are sent and data signals are accepted through the driver and interface circuits. 

All the modules mentioned above communicate with each other through Advanced Microcontroller Bus Architecture (AMBA), which is a communication protocol for data and instructions [17]. AMBA integrates Reduced Instruction Set Computing (RISC) processors into other intellectual property (IP) cores and peripherals. The 2.0 version of the AMBA standard [17] defines three sets of buses: Advance High-performance Bus (AHB), Advanced System Bus (ASB), and Advanced Peripheral Bus (APB). In the proposed design, the AHB communicates directly with the processor, and the APB communicates with the processor via the AHB. Thus, modules mounted on the AHB have faster processor access speeds, and modules on the APB are used to communicate with low-speed external devices.

**Figure 2:**
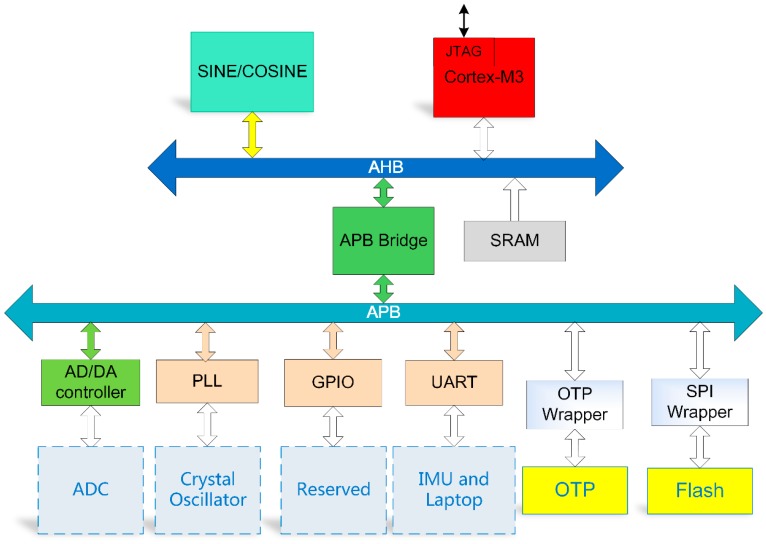
Detailed architecture of the proposed SoC.

Figure 2 shows the main modules mounted on the bus. Excluding the clock signal from the crystal oscillator, the input signals and output signals of the chip are digital. For modules on the bus, the sine and cosine calculation module, Static Random-Access Memory (SRAM), and the Cortex-M3 processor core are mounted on the AHB. The programs and data during the running of the processor are stored in the SRAM. In the design, the size of an SRAM is 8 KB with a data bit width of 8 bit and a memory depth of 8192. In addition, 16 SRAM are mounted on the AHB, and the total size is 128 KB. All modules mounted on the AHB are frequently accessed, and have faster read and write speeds than modules on the APB.

The APB Bridge module implements data conversion between the AHB interface data and the APB interface and is used to implement the module to transfer data to the AHB through the APB interface, which is then accessed by the processor.

The AD/DA controller module can provide control signals for Analog-to-Digital Converter (ADC) and Digital-to-Analog Converter (DAC) and receive data from off chip ADC or send data to off chip DAC. This module is designed to provide a universal sensor control interface and enable the system to collect more types of sensor data. For example, temperature sensor data can be obtained by the system via ADC and the AD/DA controller to perform temperature compensation of an IMU. The signal timing of the AD/DA controller is designed according to the timing requirements of the AD7690 [18] and DAC8812 [19].

A digital-analog hybrid Phase Locked Loop (PLL) receives the clock signal from the crystal oscillator and outputs a clock of a different frequency through configurable parameters for driving the digital system. This design can realize the program configuration of the system frequency so as to achieve different operating frequencies under different computing requirements. In addition, 16 General Purpose Input Output (GPIO) interfaces are reserved; 8 serial Universal Asynchronous Receiver/Transmitter (UART) modules are mounted on the APB for data transmission with the IMU and other devices such as computers. For the IMU being developed in our laboratory, the design of the 8 UART modules is to consider the use of 6 UART modules for angular velocity and angular velocity acquisition, and the other two UART modules are used for data transmission with laptops and other devices. For other interested users, different data collection methods can be used.

In addition to SRAM, the system also provides two modules for supporting data reading and writing from One Time Programmable (OTP) memory and Flash memory, which gives the system a variety of ways to start and debug. OTP memory is on-chip with a total size of 128 KB and Flash memory is off chip with a size depending on demands.

### 2.2. Function Description of the Entire SoC and the Dedicated Modules

The function of the entire system mainly includes power-on behavior, the use of dedicated modules, and the reading and writing of interface data and the configuration of clock frequency, which includes the scheme for executing the program, how the dedicated module implements hardware acceleration, how the entire system uses the sensor data to perform the corresponding functions, and the way to realize low power consumption by adjusting the clock frequency.

**Figure 3:**
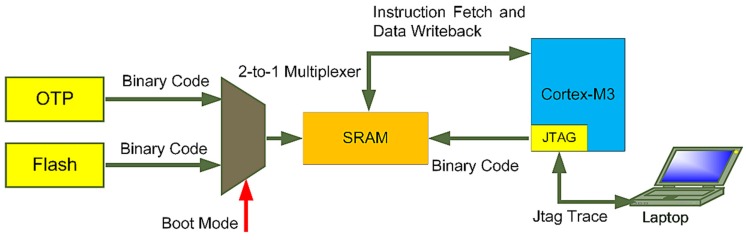
Description of the program loading process.

When the system starts, it needs to load and execute the program. The loading of the program is to write the compiled program to the SRAM and execute the corresponding instructions through the processor. The instructions are written from the memory to the SRAM by the boot loader at power-on. As shown in Figure 3, in the test phase, the program in the off-chip FLASH memory can be written into the SRAM through the Serial Peripheral Interface (SPI); when the program is solidified, the OTP write program can be executed, and the algorithm and parameters are written into the OTP memory. Because OTP memory is on-chip and is only programmable once, it increases system integration and code security. Since the system includes the Joint Test Action Group (JATG), it is supported to refresh the program in the SRAM during the running process, which facilitates the debugging of the algorithm.

The dedicated calculation module calculates the sine and cosine of the angle. The general on-chip processor does not include the sine and cosine calculation. Therefore, it is necessary to introduce a third-party library file into the program for calculation, which means that the program executes more instructions. The designed special calculation module is mounted on the AHB, and the data can be written to the address where the angle value is located in one instruction, and the second instruction can read the calculation result through the address of the output result. This calculation consists of two instructions and can be completed in one system clock cycle. Compared with the sine and cosine calculation of software algorithms, it has high execution speed and efficiency. In the navigation algorithm, since sine and cosine calculations often occur, using the dedicated module for hardware acceleration can improve the execution speed of the whole algorithm, thereby reducing the system clock requirement and further reducing the system power consumption.

In terms of interface circuits, the AD/DA controller and 8 UART modules can support data transmission of sensors such as IMU and communication with laptops.

In addition, the PLL is used to generate clock signals, and the clock frequency configuration is achieved through digital signals. Digital signals can be modified numerically by software programs.

## 3. Implementation Details of the Designed SoC

This section will introduce the implementation details of some of the modules in the system, including the PLL, sine and cosine calculation module, and the design of the bootloader. Other modules, such as bus interfaces, AD/DA controllers, etc., can be implemented using conventional design methods depending on the design requirements. 

### 3.1. Implementation Details of the PLL

The average dynamic power consumption of the circuit is proportional to the clock frequency, so reducing the clock frequency is a means of low power consumption when the calculation requirements are met. Therefore, the realization of a flexible and variable clock is helpful for the realization of low power consumption under different application conditions. For this purpose, a digitally controllable PLL is designed and implemented. This module allows users to control the system clock through the program running in the processor core, so as to dynamically implement low-power applications that meet computing requirements.

**Figure 4:**
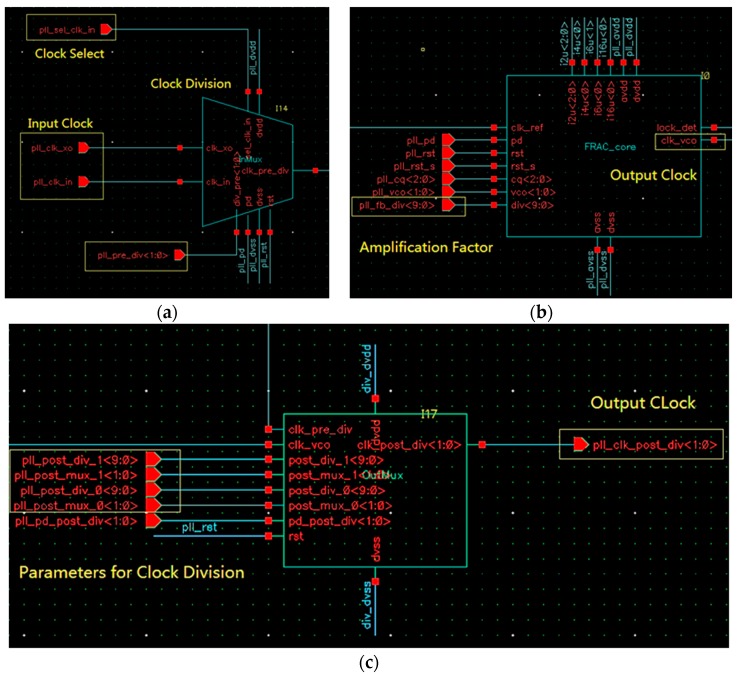
Schematic diagram of the digital-analog mixed Phase Locked Loop (PLL). (**a**) Clock selection and frequency division; (**b**) analog PLL module; (**c**) frequency division and clock output.

The PLL is a digital-analog mixed module, which converts the output clock of the crystal oscillator to a stable clock signal and the output of a high-frequency clock can be realized [20]. Its schematic diagram is shown in Figure 4. Figure 4a,c shows digital sections for implementing parameter configuration of the PLL, and Figure 4b shows an analog section for outputting a high-frequency clock.

Figure 4a shows that the PLL module supports dual clock input, and the module input clock is selected through a 2-to-1 multiplexer, and the output is divided. The division factor can be read and written by the APB, thus realizing the dynamic adjustable function of the input frequency range. In addition, the dual clock input can support the system to use the crystal clock and also use an external clock. The input clock frequency is defined as $f_{in}$, the division factor is defined as $Div_{pre}$, and the divided clock frequency is defined as $f_{pre}$, then $f_{pre}=f_{in}/Div_{pre}$. In Figure 4a, $f_{in}$ is one of the clock pll_clock_xo and the clock pll_clock_in. $Div_{pre}$ is decided by the digital port pll_pre_div.

Figure 4b shows the symbol of the analog PLL. It consists of a phase detector (PD), a loop pass filter (LPF), and a voltage-controlled oscillator (VCO) [20]. The PD is also called a phase comparator. Its function is to detect the phase difference between the input signal and the output signal and convert the detected phase difference signal into a voltage signal. The LPF is used to remove high-frequency noise, and a passive RC filter is used in the design. The VCO is used to generate a voltage-controlled clock signal. The output clock of this part is $f_{vco}$, and its frequency amplification is controlled by digital port pll_fb_div. When the value of the digital port pll_fb_div is Amp, $f_{vco}=f_{pre}\times Amp$.

Figure 4c is another clock divider module that outputs the down-converted clock (the frequency is defined as $f_{pll}$) based on the values of digital ports pll_post_div and pll_post_mux. When the values of the two ports are $Div_{post}$ and $Div_{mux}$, respectively, $f_{pll}=\frac{f_{vco}}{Div_{post}\times Div_{mux}}\;$.

According to the above description, the frequency of the output clock has the following relationship with the frequency of the input clock:

(1) $$f_{pll}=\frac{f_{in}}{Div_{pre}}\times Amp\times \frac{1}{Div_{post}\times Div_{post}}\prime$$

where the parameters $Div_{pre}$, Amp, $Div_{post}$, and $Div_{post}$ are digital and can be read and written by APB, and thus can be controlled by the program. Four adjustable parameters provide a large range of variation for the frequency of the output clock.

### 3.2. Design of Sine and Cosine Calculation Module

The Cortex-M3 processor itself does not have the ability to calculate sine and cosine values. The software implementation of this function has a large time delay and a slow calculation speed. Since sine and cosine calculations often occur in some applications, hardware circuits are used to speed up the calculations. The design principle of this module is based on look-up table method and the linear interpolation method. The data processed in the digital system is a 32-bit fixed-point number, for the input 32-bit angle value, the upper 11 bits are used for table lookup, and the lower 21 bits are used for interpolation calculation. 

When the angle value of 0 to 360 degrees is quantized by 32-bit fixed point, it is assumed that the angle indicated by the upper 11 bits is x, and the angle indicated by the lower 21 bits is y, the value of the input angle can be expressed as x+y, and the following equation is established:

(2) sin(x+y)=sin(x)·cos(y)+cos(x)·sin(y),cos(x+y)=cos(x)·cos(y)−sin(x)·sin(y).

When y is close to 0, cos(y) is approximately equal to 1, and sin(y) is approximately equal to y. The sine and cosine values of the angle can be calculated using the following expression:

(3) sin(x+y)≈sin(x)+cos(x)·y,cos(x+y)≈cos(x)−sin(x)·y.

Equation (3) is the principle on which sine and cosine calculations are based. The calculation of sin(x) and cos(x) uses the look-up table method. Since x is 11 bits, a sine lookup table of size $2^{11}\times 32$ bits and a cosine lookup table of the same size are established. The calculation of sin(x+y) and cos(x+y) is based on linear interpolation of the values obtained by the lookup tables.

**Figure 5:**
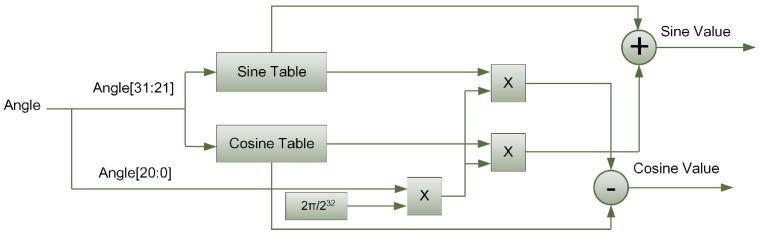
Data path of sine and cosine calculation module.

For the design scheme based on Equation (3), the schematic diagram of the data path is shown in Figure 5. In the process of interpolation calculation, the angle value corresponding to y is $y/\left(2^{32}-1\right)\times 360$ degrees, which is converted into radians as $\frac{y}{2^{32}-1}\times \frac{360}{180}\times \pi =\frac{y}{2^{32}-1}\times 2\pi \approx y\times 2\pi /2^{32}$. Dividing by $2^{32}$ is a 32-bit right shift in the circuit, which is simple to operate and avoids the use of a divider.

The calculation error of this scheme can be expressed by the following equations:

(4) $$err_{sin}=\sin\left(x+y\right)-\left[\sin\left(x\right)+\cos\left(x\right)\cdot y\right]=\sin\left(x\right)\cdot \left(1-\cos\left(y\right)\right)+\cos\left(x\right)\cdot \left(\sin\left(y\right)-y\right),\;err_{cos}=\cos\left(x+y\right)-\left[\cos\left(x\right)-\sin\left(x\right)\cdot y\right]=\cos\left(x\right)\cdot \left(\cos\left(y\right)-1\right)+\sin\left(x\right)\cdot \left(y-\sin\left(y\right)\right).$$

Using Taylor’s formula [21] for sine and cosine functions, the following equations are established:

(5) $$\sin\left(y\right)=y-\frac{y^{3}}{6}+o\left(y^{4}\right),\;\cos\left(y\right)=1-\frac{y^{2}}{2}+o\left(y^{3}\right).$$

where $o\left(y^{N}\right)$ is the higher-order infinitesimal of $y^{N}$. Substituting Equation (4) into Equation (5), the following expressions can be obtained:

(6) $$err_{sin}=\frac{1}{2}\cdot \sin\left(x\right)\cdot y^{2}-\cos\left(x\right)\cdot \frac{y^{3}}{6}+o\left(y^{3}\right),\;err_{cos}=-\frac{1}{2}\cdot \cos\left(x\right)\cdot y^{2}+\sin\left(x\right)\cdot \frac{y^{3}}{6}+o\left(y^{3}\right).$$

y is 21-bit data, which represents a maximum angle of $\frac{2^{21}-1}{2^{32}-1}\times 360\times \frac{\pi }{180}=3.07\times 10^{-3}$. Therefore, $y_{max}=3.07\times 10^{-3}$, and the maximum calculation error of this scheme can be obtained as:

(7) $$\left|err_{\sin}\right|\leq \;\frac{1}{2}\cdot \left|\sin\left(x\right)\cdot y^{2}\right|+\left|\cos\left(x\right)\cdot \frac{y^{3}}{6}\right|\leq \frac{1}{2}\cdot \left|y_{max}^{2}\right|+\frac{1}{6}\cdot \left|y_{max}^{2}\right|<5\times 10^{-6}\;,\;\left|err_{\cos}\right|\leq \;\frac{1}{2}\cdot \left|\cos\left(x\right)\cdot y^{2}\right|+\left|\sin\left(x\right)\cdot \frac{y^{3}}{6}\right|\leq \frac{1}{2}\cdot \left|y_{max}^{2}\right|+\frac{1}{6}\cdot \left|y_{max}^{2}\right|<5\times 10^{-6}.$$

**Figure 6:**
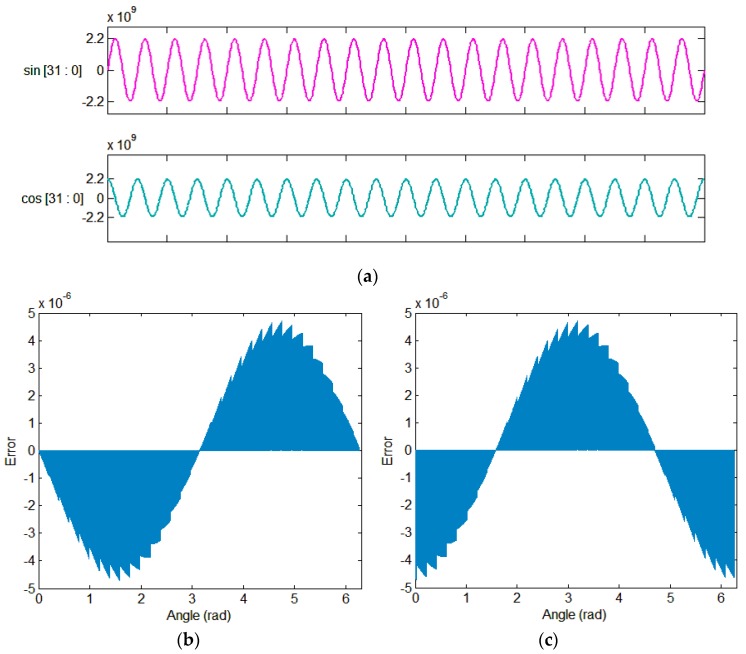
Calculation error of sine and cosine generators. (**a**) Simulated waveform of sine and cosine signals; (**b**) calculation error for sine values; (**c**) calculation error for sine values.

Based on Equation (3), the module is designed using Verilog hardware description language [22]. As shown in Figure 6, the calculation error of the sine cosine functions is obtained In VCS simulation tool [23]. The results of functional simulation show that the calculation error is less than $5\times 10^{-6}$, which is consistent with the theoretical analysis.

### 3.3. Bootloader and Its Working Principle

The bootloader module is designed to meet different application requirements, and the design content is to provide different program loading methods. For embedded SoC, the Joint Test Action Group (JTAG) debugger is supported to facilitate program debugging. This is one of the program-loading schemes, and the design method is to add a JTAG interface module into the processor core. In practical applications, programs need to be loaded from memory on-chip or off-chip. On-chip memory has higher integration, and the OPT supported by the tape out process has a write-once function, which can improve code security. The off-chip memory communicates via SPI. The interface is designed with W25Q64 FLASH as an example. The code can be read and written multiple times, which improves flexibility but reduces integration.

**Figure 7:**
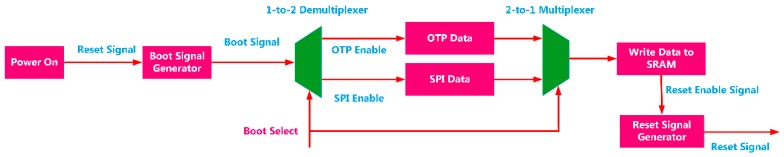
Working principle of the bootloader.

The working principle of the bootloader module is shown in Figure 7. After the power is turned on, a boot signal is generated by the reset signal. When the boot signal is effective, the reset signal is masked, and the enable signals of the OTP wrapper module and the SPI wrapper module are assigned according to the boot selection signal. The selection signal of the boot mode is 1 bit of external input and then the data in the memory is read out and written into SRAM. The data reading process of OTP and FLAH is continuously providing the address and obtaining the data according to the timing requirements. After the SRAM writing is completed, the boot signal fails and a reset signal is generated to reset other modules in the system.

## 4. Performance Results of the Chip and Application Experiments

### 4.1. Performance Test Results of the Chip

**Figure 8:**
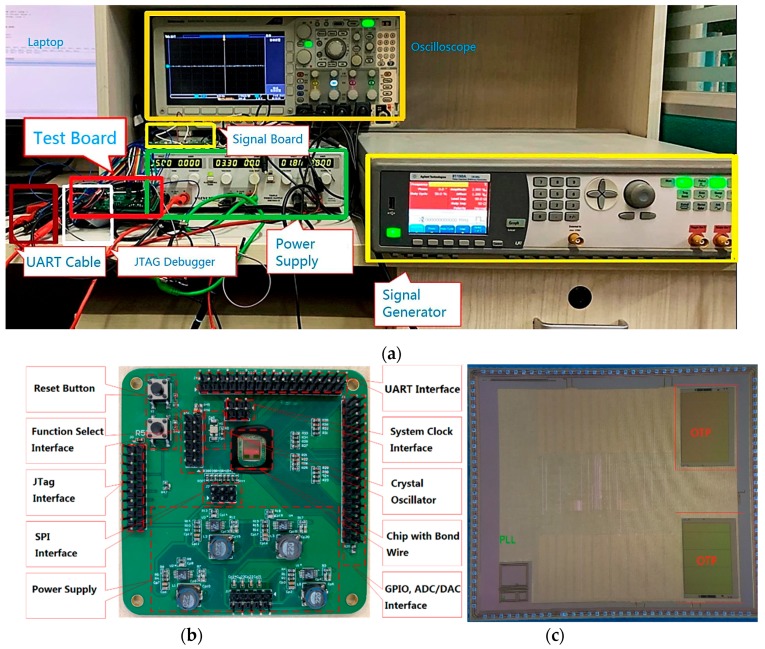
The designed chips and test equipment. (**a**) Performance test equipment and test site; (**b**) printed circuit board (PCB) for chip performance test; (**c**) die picture of the SoC.

After the SoC design was completed, the chip was taped out under the SMIC 180 nm CMOS technology. The die picture of the chip is shown in Figure 8c and the OTP and PLL modules can be distinguished. Additionally, a printed circuit board (PCB) was designed for performance testing. The test board is shown in Figure 8b and is used to provide signals and necessary interfaces. The entire test site is shown in Figure 8a. The power supply is used to supply power, and the signal generator is used to generate test signals. The signal board contains an AD7690 chip and a DAC8812 chip to test the function of the AD/DA driver. The internal programs and data can be read and written through the UART cable and JTAG debugger, and the data can be further analyzed on the laptop. The test of PLL is to continuously adjust the program to change the system driving clock and observe the clock signal through an oscilloscope. The test for Flash and OPT was completed by reading and writing to the specified address. The experimental process verified that the program can be loaded through JTAG, FLASH, and OTP. The functions of the URAT and AD/DA controllers have also been verified. In the verification of the sine and cosine calculation module, different data was written to the address corresponding to the angle in the program running in the processor, and the data was read from the address corresponding to the calculation result, and sent to the computer through the UART. The function of the module was verified, and the error curve is consistent with the simulation in Figure 6.

**Figure 9:**
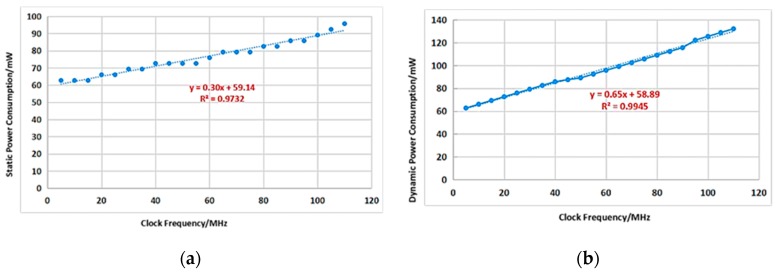
Power consumption vs. clock frequency. (**a**) Static power consumption vs. clock frequency; (**b**) dynamic power consumption vs. clock frequency.

Regarding the parameters of the chip, the total area of the chip is $5.43\times 6.25=33.94{\;\mathrm{mm}}^{2}$. By running the processor performance evaluation programs [24,25], the processor performance is obtained as 1.36 DMIPS/MHz and 3.93 CoreMark/MHz. In addition, the chip’s maximum operating frequency of 105 MHz was obtained from the experiment. In terms of power consumption evaluation, the power supply voltage of the chip is 3.3 V (3.3 V powers the UART and 1.8 V powers the digital chip and the PLL), and the power consumption of the chip under no-load (no running program) and load (performing performance evaluation program) is measured. The measurement results are shown in Figure 9, which show that the dynamic and static power consumption are 0.65 mW/MHz and 0.30 mW/MHz, respectively.

When the clock frequency is 25 MHz, the static power consumption and dynamic power consumption of the chip are 76mW and 66mW, respectively. The frequency of 25 MHz is also the clock frequency during the experiment.

In terms of size comparison, several integrated circuit solutions listed in [2] are implemented on circular PCBs, including FPGAs and other processing circuits, and the diameter of the signal processing board is less than 40 mm. The circuit scheme is much larger than the designed chip in size, but it has a DSP on the board and is stronger in computing power than the designed chip. The solutions in [10,12,13,14] are implemented based on FPGA. We will also implement the design on an FPGA [26]. Its area mainly depends on the size of FPGA. The chip size required for the implementation in [26] is $12\times 12=144{\;\mathrm{mm}}^{2}$. In general, because the circuits implemented by FPGAs will be redundant, the size of FPGA solutions will be larger than that of ASICs under the same design scheme.

In terms of power consumption, because power consumption is related to the program and hardware configuration executed, we compared the differences between chip implementation and FPGA implementation under the same design and program. When the design was implemented at the FPGA level [26], its dynamic power and static dynamic power are 634 mW and 520 mW, respectively. Compared with the FPGA-level implementation, the SoC solution implemented at the chip level has significantly improved system integration and power consumption.

### 4.2. Application Experiment

**Figure 10:**
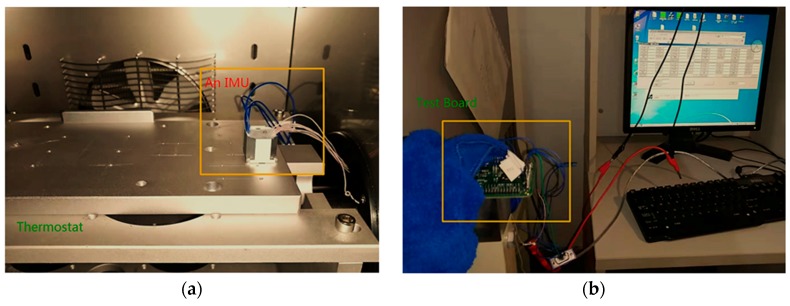
Equipment for the application experiment. (**a**) One IMU in the thermostat; (**b**) acquisition test board and the laptop for data processing.

A temperature compensation experiment was performed to verify whether the chip works. The experimental equipment is shown in Figure 10. An IMU [27] is placed in a thermostat. The temperature of the thermostat ranges from −45 °C to 85 °C. The output bias of the IMU at different temperatures was collected during the experimental process. The IMU [27] in the experiment has already contained a digital processing circuit in it, the control interface and the data output interface are SPI, and data is transmitted to the computer through the UART interface.

**Figure 11:**
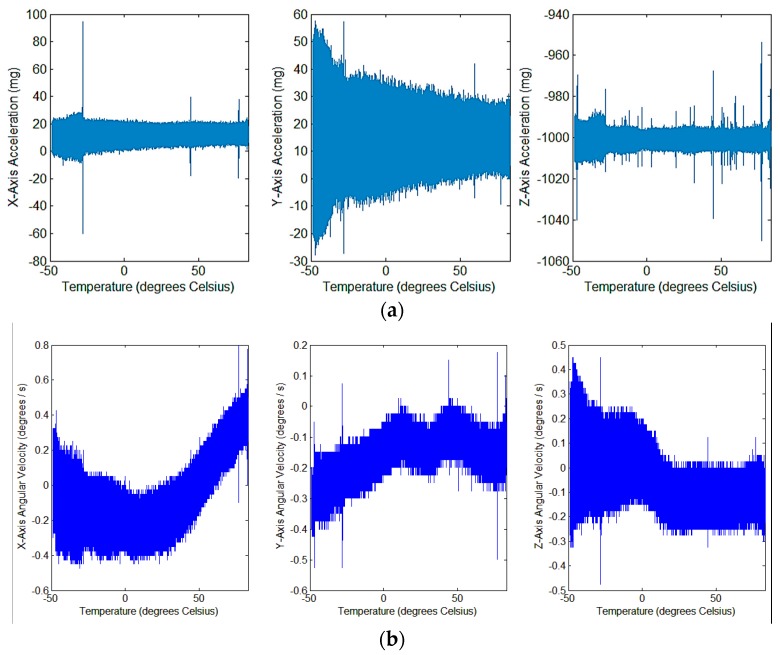
Relation curve between zero deviation of the IMU output signal and temperature. (**a**) Output of triaxial acceleration; (**b**) output of triaxial angular velocity.

A total of 228,4668 data samples were collected in the experiment, and the results are shown in Figure 11. The output of acceleration is relatively stable, and the temperature has a large effect on the output of angular velocity, which needs to be compensated.

**Figure 12:**
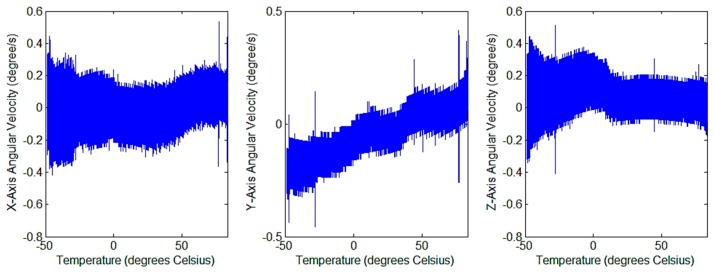
Relationship between angular velocity output after compensation and temperature.

The angular velocity compensation model uses a second-order polynomial model. The temperature is defined as t, and the angular velocity output is y; then, the output after compensation is $\hat{y}$. Then, the following equation is established:

(8) $$\hat{y}=a\cdot t^{2}+b\cdot t+c+y.$$

where a, b, and c are parameters of the curve fitting. The above compensation model was used to run a compensation program in the processor. The uncompensated and compensated angular velocity output values were collected at the same time. The output result of the uncompensated angular velocity is shown in Figure 11b, and the output result of the compensated angular velocity is shown in Figure 12.

The measurement indicators of the output bias are the average value, the maximum value of the absolute value and the standard deviation. The results before and after compensation are summarized in Table 1. From the experimental results, the execution of the compensation program has an impact on the output results, reducing the maximum zero offset of the *x* axis and *y* axis. After compensation, the standard deviation of the zero-bias variance of the *z* axis decreases, but the maximum values of the mean and absolute values increase.

This application experiment used a common polynomial-based temperature compensation scheme to verify whether the designed chip can meet the functional and computing requirements of practical applications. According to the experimental results, although the algorithm’s temperature compensation effect is limited, the designed chip does work in practical applications.

## 5. Conclusions

This article reports the design and test results of a SoC for data acquisition and processing of the MEMS IMU. The reported chip has the characteristics of miniaturization and low power consumption. Benefiting from an on-chip processor and rich interfaces and on-chip modules, the chip has high flexibility in use and can meet different usage scenarios. The on-chip dedicated calculation module can speed up the sine and cosine calculation commonly used in navigation algorithms, and the on-chip configurable clock allows users to configure clock frequency to balance the processing speed and low power consumption according to actual needs. The temperature compensation experiment further verified that the designed SoC for the MEMS IMU works in practical applications.

This chip retains many interfaces and functions and retains large on-chip memory to improve system flexibility, and it is difficult to achieve its best in terms of low power consumption. Subsequent research will be based on this content to design more specialized SoC for inertial devices (gyro, accelerometer, IMU, etc.) developed by the laboratory. Streamlined structures and hardware acceleration for signal processing algorithms for specific applications will also be developed based on this research content.

## Figures and Tables

**Table 1:** Triaxial angular velocity zero offset results before and after compensation.

Items	x Axis	y Axis	z Axis
Mean Value Before Compensation (degree/s)	0.0017	−0.1774	−0.0412
Mean Value After Compensation (degree/s)	0.0074	−0.0514	0.0647
Maximum Absolute Value Before Compensation (degree/s)	0.8000	0.5250	0.4750
Maximum Absolute Value After Compensation (degree/s)	0.5348	0.4576	0.5142
Standard Deviation Before Compensation	0.4670	0.2668	0.4750
Standard Deviation After Compensation	0.2969	0.3432	0.3091
